# Microbial Diversity of Bacteria Involved in Biomineralization Processes in Mine-Impacted Freshwaters

**DOI:** 10.3389/fmicb.2021.778199

**Published:** 2021-11-22

**Authors:** Patrizia Paganin, Chiara Alisi, Elisabetta Dore, Dario Fancello, Pier Andrea Marras, Daniela Medas, Maria Rita Montereali, Stefano Naitza, Nicola Rigonat, Anna Rosa Sprocati, Flavia Tasso, Salvatore Vacca, Giovanni De Giudici

**Affiliations:** ^1^Territorial and Production Systems Sustainability Department, Italian National Agency for New Technologies, Energy and Sustainable Economic Development (ENEA), Rome, Italy; ^2^Department of Chemical and Geological Sciences, University of Cagliari, Cittadella Universitaria, Cagliari, Italy

**Keywords:** bioremediation, sulfate-reducing bacteria, heavy metals, biomineralization, mine waters, microbial diversity, next generation sequencing, Zn sulfide precipitates

## Abstract

In order to increase the knowledge about geo-bio interactions in extreme metal-polluted mine waters, we combined microbiological, mineralogical, and geochemical analyses to study the indigenous sulfate-reducing bacteria (SRB) involved in the heavy metal (HM) biomineralization processes occurring in Iglesiente and Arburese districts (SW Sardinia, Italy). Anaerobic cultures from sediments of two different mining-affected streams of this regional framework were enriched and analyzed by 16S rRNA next-generation sequencing (NGS) technique, showing sequences closely related to SRB classified in taxa typical of environments with high concentrations of metals (*Desulfovibrionaceae*, *Desulfosporosinus*). Nevertheless, the most abundant genera found in our samples did not belong to the traditional SRB groups (i.e., *Rahnella*, *Acinetobacter*). The bio-precipitation process mediated by these selected cultures was assessed by anaerobic batch tests performed with polluted river water showing a dramatic (more than 97%) Zn decrease. Scanning electron microscopy (SEM) analysis revealed the occurrence of Zn sulfide with tubular morphology, suggesting a bacteria-mediated bio-precipitation. The inocula represent two distinct communities of microorganisms, each adapted to peculiar environmental conditions. However, both the communities were able to use pollutants in their metabolism and tolerating HMs by detoxification mechanisms. The Zn precipitation mediated by the different enriched cultures suggests that SRB inocula selected in this study have great potentialities for the development of biotechnological techniques to reduce contaminant dispersion and for metal recovery.

## Introduction

Abandoned metal(loid)-sulfide mines are a worldwide environmental concern spreading potentially toxic pollutants through soils, waters, and eventually the whole ecosystem. Mine drainages, in particular acid mine drainages, are commonly characterized by high concentrations of toxic metals and sulfate and represent a risk on involved freshwater ecosystem, causing contamination and chemistry changes of water courses (De Giudici et al., 2014, 2018; Hwang and Jho, 2018; Rodrigues et al., 2020). Such waters represent an extreme environment for life but also a unique ecological niche for metabolically active acidophilic bacteria that are well adapted to the multiple environmental stresses encountered. Some of these microorganisms directly respond to contamination and favor natural metal attenuation through direct control of biomineralization processes, or favoring compartmentalized response to stress, and then the evolution of the system itself (Lawrence et al., 1998; De Giudici et al., 2017; van der Graaf et al., 2020; Newsome et al., 2021).

In the last decade, the interest in studying microbial consortia and their role in mineralogical and geochemical processes has dramatically increased (Al-Shayeb et al., 2020; Arbour et al., 2020; Gionfriddo et al., 2020). In such polluted environments, the characterization of microenvironments and their specific microorganism community is a key to understanding the impacts of mine drainage on microbial ecology and evolution and to recognizing metal-tolerant bacteria that may have bioremediation properties. The use of NGS and metagenomic tools significantly improves the ability to identify bacterial taxa and to quantify bacterial abundance and diversity in polluted environments. This allows to correlate the bacterial community structure and functional characteristics to metal immobilization processes. Recent studies showed that, in rivers where the sedimentation regime prevails, biogeochemical barriers can naturally develop and can reduce metal mobility and dispersion (Shumilin et al., 1993; Baltrėnas and Baltrėnaitė, 2020; Dore et al., 2020) by means of the precipitation of secondary supergene sulfides, promoted by sulfate-reducing bacteria (SRB). Eventually, the effort to understand the resiliency processes will provide remediation tools more cost effective and sustainable.

For many years now, the approach known as anaerobic bioremediation using SRB is considered a promising process, alternative to traditional chemical methods, for treating rivers affected by mining activities. SRB can degrade many different substrates to reduce sulfate to hydrogen sulfide (Sánchez-Andrea et al., 2012), preferring simple organic compounds or hydrogen as electron donors. The hydrogen sulfide produced reacts with dissolved metal ions forming low soluble metal sulfide precipitates (Rodrigues et al., 2019). Thus, by sulfate reduction, metals can be precipitated, recovered, and reused.

Anyway, the threshold of tolerance of bacteria to high concentrations of heavy metals is one of the main factors limiting their use in bioremediation. Although the recent literature expanded the Zn concentration limits, toxic to sulfate reducers, from 210 mg/l (Radhika et al., 2006) up to 450 mg/l (Sánchez-España et al., 2020), the physicochemical limits enabling SRB to be active are still under study. For instance, van der Graaf et al. (2020) suggested that in the acidic La Zarza pit lake, with Zn concentrations exceeding 500 mg/l, elemental sulfur reduction and disproportionation of S_8_^0^ are the dominant processes, while the sulfate reduction starts at a later stage of the natural bioremediation, when the metal attenuation is already triggered.

For this reason, the identification of SRB strains resistant to high concentrations of Zn is crucial for the development of effective bioremediation interventions to be implemented in waters highly polluted by this metal.

Another key point for the effectiveness of this kind of bioremediation strategy is the synergistic interaction among microbial species: several studies highlighted the dependence of SRB on other microorganisms, like fermentative bacteria (Martins et al., 2011; Rodrigues et al., 2019). Since complex microbial communities are responsible for sulfate reduction processes, the appropriate selection of the inoculum to be used in bioremediation activity is essential.

In the nineteenth and twentieth centuries, mining activity linked to Pb and Zn production represented the main economic activity in the Iglesiente and Arburese mine districts (SW Sardinia, Italy). In the aftermath of mining closures, a profound impact on the environment was left behind, still needing remediation. Rio Irvi is the most polluted river of the area with contents of metals (Zn, Pb, Fe, etc.) two to three orders of magnitude greater than other mine-affected rivers of Sardinia, such as Rio Naracauli and Rio San Giorgio (De Giudici et al., 2018). Interestingly, mineralogical and geochemical analyses performed on core sediments of these two rivers having the lower metal loads showed the presence of secondary sulfides whose precipitation is presumably mediated by SRB (De Giudici et al., 2017; Dore et al., 2020). On the contrary, in Rio Irvi (showing negligible sediment thickness) evidence of processes leading the abatement of metal contents was not revealed (De Giudici et al., 2018). Based on these data, De Giudici et al. (2018) suggested that induction of metal load attenuation by microbially driven precipitation, as observed in sediments of Rio Naracauli and Rio San Giorgio, could be a possible remediation strategy also for Rio Irvi water.

With the aim to reproduce the mineralization processes of sulfide bio-precipitation observed in Rio Naracauli and Rio San Giorgio, sediments of these streams were collected and used to select microbial inocula suitable for the treatment of the extremely Zn-contaminated Rio Irvi water. To this end, the sulfate and Zn removal potential of these selected consortia was tested in batch experiments. Since the performance of the bio-precipitation process is highly dependent on interactions among microbial populations, the structure of the bacterial communities was deeply investigated and compared by the NGS technique.

## Materials and Methods

### Study Area, Core Sampling, and Analysis

**FIGURE 1:**
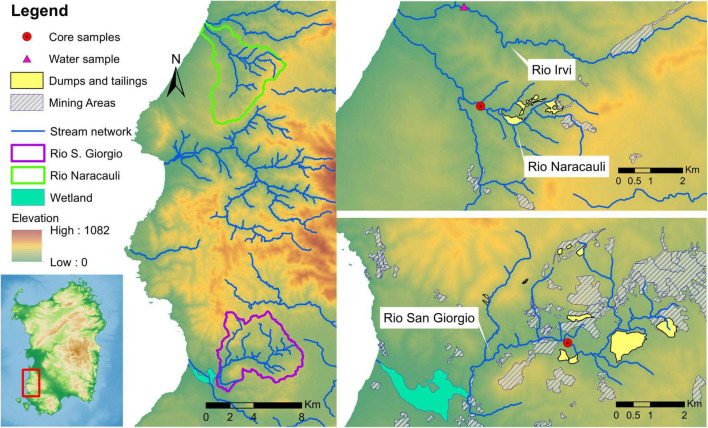
Schematic maps of study area and location of collected samples.

Three contaminated streams flowing in mining areas were involved in the present study: Rio Irvi (Frau et al., 2015; De Giudici et al., 2018) and Rio Naracauli (De Giudici et al., 2014), which flow in the Montevecchio-Ingurtosu district, and Rio San Giorgio (De Giudici et al., 2017), which flows in the Iglesias district (Figure 1). Discharge (L/s) and concentration ranges of selected chemical species of waters from Rio Naracauli, Rio San Giorgio, and Rio Irvi are shown in Supplementary Table 1. Rio San Giorgio and Rio Irvi are characterized by very similar discharge values, ranging between 20 and 40 L/s, whereas along Rio Naracauli, discharge shows progressive and sharp increments from 0.4 to 35 L/s. Waters from Rio Naracauli and Rio San Giorgio have near-neutral to slightly alkaline pH values (pH 7.6–8.4 and 7.7–8.3, respectively). Lower pH values were observed (De Giudici et al., 2018) along Rio Irvi (pH 4.8–6.3). Zinc is the most abundant metal, and it reaches the highest concentrations along Rio Irvi (760–860 mg/l). Iron tends to be lower than the DL (detection limit) along Rio Naracauli and Rio San Giorgio, and it is found in significant amounts (Fe 130–220 mg/l) in Rio Irvi water. Other metals characterized by relevant concentrations are Mn, Cd, Pb, Ni, and Co, which reach the highest values along the Rio Irvi (up to 66 mg/l, 2,000 μg/l, 440 μg/l, 3,100 μg/l, and 1,700 μg/l, respectively). Sulfate concentrations show the highest values along Rio Irvi (up to 3,400 mg/l), and similar values along Rio Naracauli (290–850 mg/l) and Rio San Giorgio (230–620 mg/l) (De Giudici et al., 2014, 2017, 2018). The Zn load measured in these rivers differs up to three orders of magnitude: these differences can be attributable to the kind of pollution sources and the presence (or absence) of mineralogical and biogeochemical processes in the hyporheic zone (Dore et al., 2020).

To reproduce the processes allowing the sulfide bio-precipitation observed in sediments of Rio Naracauli and Rio San Giorgio, core sample sediments were collected along their riverbeds (Figure 1) and the sampling core sites were selected taking into account the abundance of framboidal metal sulfides detected in the stream sediments in previous investigations (see De Giudici et al., 2017; Dore et al., 2020).

Core sediments from Rio Naracauli were collected downstream from the main tailing dump of the mining district (Figure 1), an area characterized by lush vegetation of *Juncus acutus* L. and *Phragmites australis* L. Undisturbed core samples of stream sediments were collected through a core sampler (Atlas Copco’s COBRA) which restored the samples in sealed plastic bags. Samples were transferred to the lab and stored at −20°C until use.

For X-ray diffraction analysis (XRD), samples from the sediment cores were dried at room temperature and lightly ground in an agate mortar. Mineralogical characterization was performed by an automated PANalytical X’Pert Pro diffractometer, on an angular range 5°–70° 2θ, operating at 40 kV and 40 mA, with Ni-filtered Cu Kα radiation (λ = 1.54060 Å) and the X’Celerator detector. Air-dried and not-ground samples were analyzed by SEM, performed by a FEI Quanta 200 equipped with a Thermo Fisher UltraDry energy-dispersive spectroscopy (EDS) detector at the CeSAR laboratory (University of Cagliari). Analyses were performed on uncoated samples under low vacuum and with variable accelerating voltage (15–25 kV) and spot size 4–6.

Chemical characterization of the core sediments used for the bacterial inocula was performed according to the EPA method 3050. A high-purity mixture of 2 ml of H_2_O_2_ (30%, Sigma-Aldrich), 4 ml of HF (40%, Chem-Lab), 9 ml of HNO_3_ (67–69%, Carlo Erba), and 2 ml of HCl (34–37%, Chem-Lab) was added to the solids (0.25 g of dried material) into microwave vessels. Acid digestion was performed by microwave ETHOS One, Advanced Microwave Digestion System, Milestone. After cooling, the mixtures were transferred into Teflon beakers and heated in a hot plate (110°–150°C) according to Medas et al. (2013). Finally, samples were filtered (0.4 μm), and the solutions were made up to 50 ml of final volume using ultrapure water (Millipore, Milli-Q©, 18.2 MΩ cm). Samples were processed together with two blank and two reference materials (SRM—Montana Soil 2710—Highly Elevated Trace Element Concentrations, and RTS-1—Sulfide Ore Mill Tailings) prepared with the same mixture. Total sulfur (Stot) and selected metal concentrations (Fe, Zn, Pb, Mn, and Cd) were analyzed by inductively coupled plasma optical emission spectrometry (ICP-OES, ARL Fisons ICP Analyzer 3520 B). Both samples and reference materials were digested and analyzed in duplicate to estimate the precision of the acid digestion (standard deviation/mean concentration): S_tot_ 0.3–4%, Fe 0.4–2.3%, Zn 0.4–3.6%, Pb < 2.3%, Mn 0.3–9.7%, and Cd 2.5–3.3%. The percentage of recovery (mean measured concentration/certified concentration of the reference materials) was calculated to evaluate the analytical accuracy of the acid digestion procedure: S_tot_ 98–100%, Fe 95–100%, Zn 97–101%, Pb 95%, Mn 96–100%. Procedural blanks and a reference solution (EnviroMAT-Drinking Water Low EP-L-3) were analyzed by ICP-OES after every five samples to estimate potential contaminations and the accuracy (0.5–4.4%) and precision (1.0–4.2%) of trace element analysis.

### Sulfate-Reducing Bacteria Inoculum Preparation and Cultivation Conditions

Samples for microbial inocula were scraped from the frozen core sediments in the portion between 150 and 165 cm in Rio Naracauli, and 64–72 cm in Rio San Giorgio. Enrichment cultures of fermentative and SRB were obtained by transferring 5 g of frozen sediment into 250-ml bottles capped with butyl rubber stoppers, containing 100 ml of sterile standard “Postgate B” liquid medium (2.0 g MgSO_4_⋅7H_2_O, 1.0 g CaSO_4_, 1.0 g NH_4_Cl, 0.5 g KH_2_PO_4_, 1.0 g yeast extract, 3.5 g sodium lactate, 0.5 g FeSO_4_⋅7H_2_O, 0.1 g thioglycolic acid, and 0.1 g ascorbic acid in 1,000 ml distilled water) (Atlas, 2005). The headspace air was replaced with O_2_-free N_2_ gas, and the cultures were incubated at 28°C on a rotary shaker at 130 rpm, until blackening (indicating H_2_S production due to the growth of SRB). Sterile controls were also set up throughout the experiments.

**FIGURE 2:**
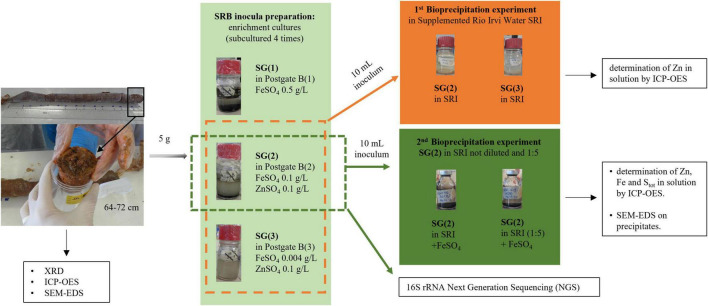
Schematic representation of experiments and analysis carried out for Rio San Giorgio sediment.

In order to test the optimal conditions for growth and metal bio-precipitation, the enrichment cultures from each sediment were transferred to fresh standard “Postgate B” liquid medium (indicated with the number “1”) and to its modified versions (“2” and “3”) supplemented with Zn and Fe sulfate at different concentrations, as described in Table 1 and Figure 2. In media 2 and 3, Zn was added to allow SRB growth and activity, according to literature data (Azabou et al., 2007; Wolicka et al., 2015). In the same media, the amount of Fe was reduced to favor Zn precipitation and to make it more visible: in medium 2, FeSO_4_ was added in the concentration suggested by Wolicka et al. (2015) in their Zn bio-precipitation experiments, and in medium 3 it was further reduced to the minimum concentration used for the preparation of other sulfate reducer culture media, as reported by Atlas (2005). For each different medium, the enrichment was repeated at least four times. Figure 2 represents the schematic diagram of experiments carried out on Rio San Giorgio sediment. The same experimental setup was applied on Rio Naracauli sediment.

**TABLE 1:** Concentrations of FeSO_4_ and ZnSO_4_ in standard (1) and modified Postgate B media (2 and 3).

Medium	FeSO_4_ (g/L)	ZnSO_4_ (g/L)
1	0.5	−
2	0.1	0.1
3	0.004	0.1

### Bio-Precipitation Experiments

#### Best-Performing Inoculum Selection

Zn sulfide bio-precipitation was evaluated in batch tests (Figure 2), using Rio Irvi water collected along the stream. In order to choose the most efficient inocula in the biological formation of Zn sulfide precipitates in Rio Irvi water, a first set of bio-precipitation experiments was carried out using bacterial consortia selected from the enrichment cultures supplemented with ZnSO_4_ and different concentrations of FeSO_4_ (conditions “2” and “3” in Table 1). Inocula from Rio San Giorgio and Rio Naracauli sediments, grown in 0.1 g/l FeSO_4_ (condition “2”), are indicated as SG(2) and N(2), respectively, while inocula selected from medium with 0.004 mg/l FeSO_4_ (condition “3”) are specified as SG(3) and N(3).

To favor microbial activity, Rio Irvi water was supplemented with the same chemicals of the standard Postgate B medium, except for Fe and Zn (SRI: Supplemented Rio Irvi water). Then, it was adjusted to pH 7.0 with 1 M NaOH solution and aliquoted in 250-ml bottles. Each aliquot (100 ml) was inoculated with 10 ml of each bacterial consortium from the aforementioned enrichment cultures. These suspensions, prepared and kept in anaerobic conditions, were incubated at 28°C and maintained on a rotary shaker at 130 rpm. A not-inoculated control was introduced to compare the Zn removal in the absence of bacteria. At the end of the experiment, the precipitates and the solutions were separated by filtration (Nuclepore polycarbonate filter 0.45 μm). Zinc was determined before and after the bio-precipitation experiments in filtered samples (0.45-μm membrane filter, Millex-HA, Millipore, Bedford, MA, United States) by ICP-OES (Perkin-Elmer Optima 2000 DV) equipped with a cyclonic spray chamber. The amount of Zn bio-removal was determined by the difference between the final concentrations of Zn in the inoculated SRI solutions compared to the control. The most efficient inocula in the sulfate reduction activity were chosen to carry out a second bio-precipitation experiment.

#### Bio-Precipitation Experiments

SG(2) and N(2) were selected as the best-performing inocula and used to carry out a second set of bio-precipitation experiments in SRI, prepared as described above, but added with FeSO_4_ (0.1 g/l) to have immediate evidence of the bio-precipitation process. The same experiment was repeated with a fivefold dilution of Rio Irvi water, in order to evaluate a possible inhibition of the inoculum performance due to the high Zn concentration (Figure 2). At the end of the experiments, the precipitates and the solutions were separated by filtration (Nuclepore polycarbonate filter 0.45 μm) and recovered to perform minero-chemical (SEM-EDS) and chemical (ICP-OES, ARL Fisons ICP Analyzer 3520 B) analyses, respectively. Procedural blanks and the reference solution EnviroMAT-Drinking Water Low EP-L-3 were analyzed by ICP-OES to estimate potential contaminations and the accuracy (<5%) and precision (<5%) of the analysis. Zn, Fe, and S_tot_ measurements in enriched Rio Irvi water sample, with and without the different inocula, were performed in duplicate, after a total acid solubilization of the samples. The total digestion of the mixtures was accomplished using a microwave-assisted acid dissolution procedure consisting in adding to each sample 7 ml of HNO_3_ 69% (TraceSELECT Ultra, Honeywell, Fluka) and 3 ml of H_2_O_2_ 30% (Suprapur, Merck) and then placing the obtained solutions in a microwave system (ETHOS EASY, Milestone) to complete the dissolution.

### Total DNA Extraction, 16S rRNA Next-Generation Sequencing, and Bioinformatics Analysis of the Best-Performing Inocula

In order to study the microbial community structure of the inocula selected as best performers in sulfate reduction activity, total DNA was extracted from the two enrichment cultures N(2) and SG(2). Each inoculum (2 ml) was centrifuged for 25 min at 12,000 *g*. The supernatant was discarded, and the pellet was washed according to Frau et al. (2015) to remove inhibitors and contaminants. The subsequent steps in the extraction were performed using the DNeasy PowerSoil kit (Qiagen, Hilden, Germany), according to the manufacturer’s protocol. DNA samples were quantified using the Thermo Fisher Scientific NanoDrop Spectrophotometer, and the DNA quality was checked by 260/280- and 260/230-nm ratios and by 0.8% agarose gel electrophoresis. 16S rRNA amplicon sequencing of the bacterial consortia was obtained with an NGS approach, using the MiSeq platform (Illumina, San Diego, CA, United States).

Aliquots of DNAs (30 ng/μl final concentration) were subjected to 16S V3-V4 rRNA gene library preparation and sequencing (Bio-Fab Research, Rome, Italy). Illumina MiSeq Sequencing was performed as described by Ruocco et al. (2021). Briefly, Illumina adapters that overhang nucleotide sequences were added to the gene-specific primer sequences targeting the V3-V4 regions. After 16S amplification, a PCR cleanup was done to purify the V3–V4 amplicon from free primers and primer-dimer species. A subsequent limited cycle amplification step was performed to add multiplexing indices and Illumina sequencing adapters by using a Nextera XT Index Kit. Finally, libraries were normalized and pooled by denoising processes and sequenced on an Illumina MiSeq Platform with 2 × 300 bp paired-end reads. Taxonomy was assigned using a “homemade” Naive Bayesian Classifier trained on V3–V4 16S sequences of the SILVA 138 database. The QIIME 2 v2020.8 (Quantitative Insights Into Microbial Ecology) platform was used for microbiome analysis from raw DNA sequencing data. The QIIME analysis workflow was performed by demultiplexing, quality filtering, chimera removal, and taxonomic assignment.

The full dataset of raw data was deposited at the NCBI database (BioProject ID: PRJNA762673).

The full PICRUSt2 pipeline was used for prediction of Enzyme Commission number (EC) abundances based on the 16S rRNA (Douglas et al., 2019). Both ASVs and biome table previously generated were used as the input. The predictions were performed with the hidden-state prediction by castor R package v1.6.5 (Louca and Doebeli, 2018) and used for inferring pathway abundances (Ye and Doak, 2009).

Diversity indices and other statistical analyses were performed in the R environment v3.6.3 (R Foundation for Statistical Computing, Vienna, Austria).

The functional count tables from PICRUSt2 were used to detect differentially abundant EC (Enzyme Commission number) by edgeR v3.26.8. A volcano plot labeling and highlighting top significant genes was used to display the results (FDR = 0.01, logFC = 6.0).

A heatmap was performed by QIIME 2 v2020.8 heatmap plug-in on each metadata category ([Zn], [Fe], [Mn], [Pb], [S], Depth) and from domain to species levels (or and from lowest to highest levels).

## Results

### Mineralogical and Chemical Characterization of Rio Naracauli and Rio San Giorgio Sediment Samples

Core sediments from Rio Naracauli were made up of an alternation of unconsolidated sand-gravel layers and consolidated clay layers, with abundant organic material (mainly roots). XRD analysis revealed the presence of quartz, feldspars, and phyllosilicates as the main crystalline phases at each depth. Also, carbonates (calcite, dolomite, siderite, ankerite, and smithsonite) and sulfides (sphalerite, pyrite, wurtzite) were detected in minor amount (Supplementary Figure 1). Core sediments from Rio San Giorgio consisted of a succession of unconsolidated reddish sand and gray silts and clays. The main detected minerals were gypsum, quartz, calcite, and dolomite. Also, pyrite, sphalerite, galena, goethite, hemimorphite, and cerussite were widely detected (De Giudici et al., 2017).

**FIGURE 3:**
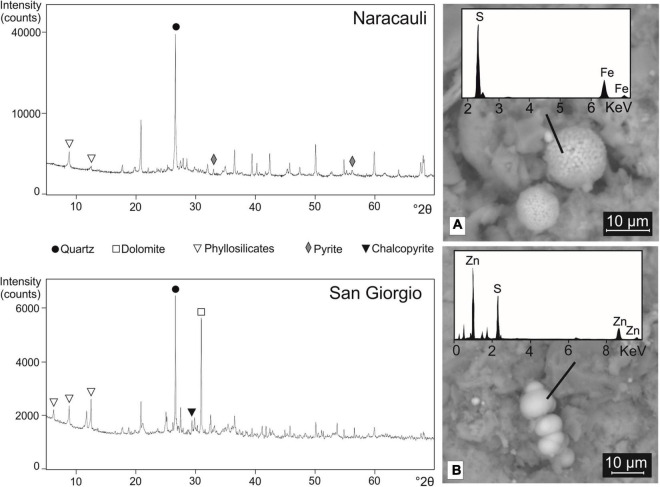
XRD patterns of sediments from Naracauli (upper) and San Giorgio (lower) core samples used for microbial inocula. On the right, scanning electron microscopy (SEM) images of sediments from Rio Naracauli showing **(A)** framboids of FeS_2_ and **(B)** ZnS.

XRD patterns of the core sediment portions selected for the microbial inocula were characterized by similar mineralogical compositions, mainly made up of quartz, phyllosilicates, and feldspars. Pyrite was detected at Naracauli whereas chalcopyrite at San Giorgio (Figure 3). In agreement with XRD results, the SEM imaging showed the presence of framboidal FeS_2_ in Rio Naracauli sediments (Figure 3A); interestingly, also the presence of framboidal sphalerite was observed (Figure 3B). These framboidal metal sulfides are not comprised within the ore primary minerals, and we attributed them to authigenic minerogenesis likely induced by SRB.

Table 2 shows the concentration ranges of significant chemical components in core sediments used in the selection of the bacterial inocula. Sediments from Rio San Giorgio showed the highest concentrations of metals, and Fe and Zn were the most abundant pollutants (134,000 and 77,200 mg/kg, respectively). Total S varied by one order of magnitude in the investigated sediments: 8,400 mg/kg at San Giorgio, and 25,910 mg/kg at Naracauli.

**TABLE 2:** Concentration of selected chemical elements in core sediments used for the bacterial inocula (i.e., the portion comprises between 150 and 165 cm in Rio Naracauli, and between 64 and 72 cm in Rio San Giorgio).

Sediment	S_tot_	Fe	Zn	Pb	Mn	Cd
	mg/kg	mg/kg	mg/kg	mg/kg	mg/kg	mg/kg
Naracauli (N)	25,910	33,000	32,000	6,390	950	300
San Giorgio (SG)	8,400	134,000	77,200	8,000	5,520	330

### Enrichment Cultures and Sulfate-Reducing Bacteria Activity

**FIGURE 4:**
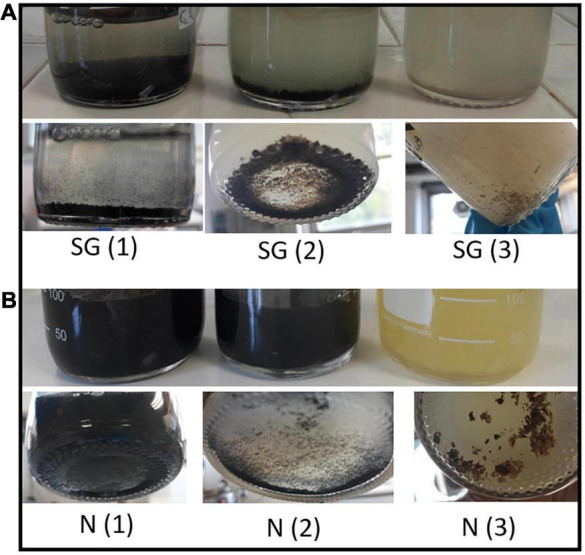
Growth of sulfate-reducing bacteria in the three different media inoculated with sediments of Rio San Giorgio **(A)** and Rio Naracauli **(B)** and details of Fe sulfide (black) and Zn sulfide (brownish) precipitates.

Both enrichment cultures (SG and N) showed bacterial growth in all the liquid media tested conditions (1, 2, and 3 in Table 1). Moreover, H_2_S formation was observed in all experiments and activity of SRB was ascertained by the formation of precipitate which appeared a few days after the inoculation of the cultures. No visual differences were observed between SG and N precipitates in the same kind of medium: in the presence of 0.1 g/l FeSO_4_ (conditions 1 and 2), inocula from the two sediments produced a black precipitate, while a white to light-brown precipitate became evident in the medium with a content of ZnSO_4_ two orders of magnitude larger than FeSO_4_ (condition 3) (Figure 4 and Table 1).

Bacteria, microbial biofilms, and mineral precipitates in liquid media were observed also by optical microscope and SEM (Supplementary Figure 2).

### Bio-Precipitation: Zn, Fe, and Sulfate Removal From Rio Irvi Water Solution

The four SRB consortia enriched in liquid media containing Zn sulfate and different Fe sulfate concentrations [N(2), SG(2), N(3), SG(3)] were tested for their Zn sulfide bio-precipitation ability in Rio Irvi water extremely rich in Zn (as shown in Table 3).

**TABLE 3:** Rio Irvi water selected chemical components at the beginning of the experiments.

	Fe	Mn	Zn	Mg	Ca	Na	K
	(mg/L)	(mg/L)	(mg/L)	(mg/L)	(mg/L)	(mg/L)	(mg/L)
	5.7	38	550	150	250	84	7.9
Surface water discharge limits (D.lgs 152/06)	≤2	≤2	≤0.5				

After 60 days from the experiment with the four SRB inocula, the Zn removal percentage in solution was higher than 97% in all the samples. In particular, the best performances were observed for the inoculum SG(2), removing 99.74% of Zn, and for N(2), reducing Zn concentration to values below the detection limit (Table 4).

**TABLE 4:** First bio-precipitation experiment: values of Zn in filtered solution samples (prepared as described in the “conditions” column) and Zn abatement (% respect to CTRL) with different inocula.

Samples	Conditions	Zn (mg/L)	Zn removal rate (%)
Rio Irvi Water	Rio Irvi water	520	
CTRL	SRI* without inoculum	180	
SG(2) Irvi	SRI* and inoculum SG(2)	0.47	**99.7%**
SG(3) Irvi	SRI* and inoculum SG(3)	1.5	**97.9%**
N(2) Irvi	SRI* and inoculum N(2)	<DL	**100%**
N(3) Irvi	SRI* and inoculum N(3)	0.70	**99.6%**

*SRI composition: 2.0 g/l MgSO_4_⋅7H_2_O, 1.0 g/l CaSO_4_, 1.0 g/l NH_4_Cl, 0.5 g/l KH_2_PO_4_, 1.0 g/l yeast extract, 3.5 g/l sodium lactate, 0.1 g/l thioglycolic acid, 0.1 g/l ascorbic acid in Rio Irvi water. DL, limit of detection, 0.03 mg/l. The values in bold are those expressed as %, to distinguish them from the concentration values.

The best-performing inocula, SG(2) and N(2), were used in a second set of bio-precipitation experiment in SRI water, this time with the addition of FeSO_4_ (0.1 g/l), in order to promptly observe the bio-precipitation process. These experiments confirmed the previous results about Zn removal and also demonstrated the ability to decrease the amount of Fe and S_tot_ in solution (Table 5). Indeed, the two inocula showed a Zn removal rate of 100% in both diluted and original water samples. The inoculum N(2) appeared to be more efficient in removing Fe in undiluted water.

**TABLE 5:** Second bio-precipitation experiment: values of S_tot_, Zn, and Fe and their abatement (% respect to CTRL) due to different inocula.

Samples	Conditions	Zn	Fe	S_tot_	Zn removal	Fe removal	S_tot_ removal
		(mg/L)	(mg/L)	(mg/L)	%	%	%
CTRL	SRI (plus 0.1 g/l FeSO_4_), undiluted; without inoculum	290	22	1,810			
SG(2) Irvi	SRI (plus 0.1 g/l FeSO_4_), undiluted; with inoculum SG(2)	<DL	6.8	820	**100%**	**68.7%**	**54.7%**
N(2) Irvi	SRI (plus 0.1 g/l FeSO_4_), undiluted; with inoculum N(2)	<DL	0.73	860	**100%**	**96.6%**	**52.3%**
CTRL (1:5)	SRI (plus 0.1 g/l FeSO_4_), diluted 1:5; without inoculum	50	37	760			
SG(2) Irvi (1:5)	SRI (plus 0.1 g/l FeSO_4_), diluted 1:5; with inoculum SG(2)	<DL	0.15	410	**100%**	**99.6%**	**46.5%**
N(2) Irvi (1:5)	SRI (plus 0.1 g/l FeSO_4_), diluted 1:5; with inoculum N(2)	0.04	0.05	460	**99.9%**	**99.9%**	**39.8%**
DL	Limit of detection	0.03	0.02	7			

*The values in bold are those expressed as %, to distinguish them from the concentration values.*

**FIGURE 5:**
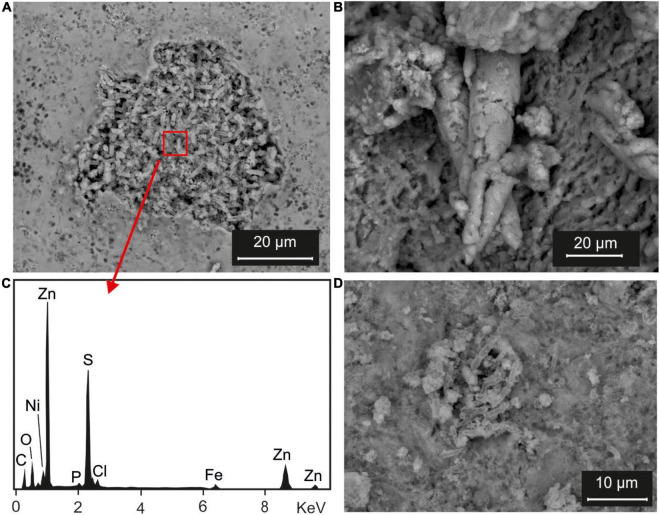
Scanning electron microscopy (SEM) analysis: **(A)** BSE images of the bioprecipitates recovered from an experiment performed with inocula selected from Rio Naracauli core sediments and Rio Irvi water [sample N(2) Irvi] and **(B)** its EDS spectrum; **(C)** BSE images of bioprecipitates recovered from an experiment performed with inocula selected from Rio Naracauli core sediments and diluted Rio Irvi water [sample N(2) Irvi(1:5)]; **(D)** BSE images of bioprecipitates recovered from an experiment performed with inocula selected from Rio San Giorgio [sample SG(2) Irvi].

Results of SEM analysis, performed on precipitates recovered at the end of experiment, showed noteworthy differences between samples with SRI diluted and not diluted. In fact, BSE (backscattered electron) images of N(2) Irvi and SG(2) Irvi samples (Figures 5A,D, respectively) clearly showed a structure with tubular morphology typical of bacterial bio-precipitates, mainly made up of S and Zn (Figure 5B).

Differently, in the BSE images of precipitates recovered after experiments with diluted SRI (Figure 5C), it was not possible to recognize structures attributable to biological activity. These samples were more homogenous with elongated structures ranging between 20 and 50 μm in length. These structures were enriched in Zn and S, with minor C, O, Mg, Si, P, Cl, K, Ca, Fe, and Mn (Supplementary Figure 3).

### Next-Generation Sequencing Results

#### Bacterial Community Structures of Best-Performing Selected Inocula N(2) and SG(2)

A total of 31,412 effective sequences with an average length of 405.27 bp were obtained from the two best-performing inocula [N(2) and SG(2)] through Illumina high-throughput sequencing technology [18,105 from N(2) and 13,307 from SG(2)]. All rarefaction curves tended to the saturation plateau, demonstrating adequate volume of sequencing data (Supplementary Figure 4). The sequences were assigned to 173 amplicon sequence variants (ASVs) with percentage of confidence ≥ 75%.

The sample N(2) hosted the greatest abundance of bacterial taxa (100 ASVs), compared to the 73 ASVs of SG(2). The only common species in the two microbial inocula was *Clostridium sensu stricto.* Nevertheless, the values of richness (Chao1) and Shannon and Simpson indexes, obtained at family, genus, and species levels, showed a very similar alpha-diversity estimation for the two samples (Table 6).

**TABLE 6:** Comparison of the estimated richness and diversity indices.

	Family level			Genus level			Species level		
	Shannon	Simpson	Chao1	Shannon	Simpson	Chao1	Shannon	Simpson	Chao1
N(2)	2.400	0.626	37	2.486	0.626	54	3.130	0.780	69
SG(2)	2.839	0.777	31	2.963	0.780	44	3.211	0.805	57

**FIGURE 6:**
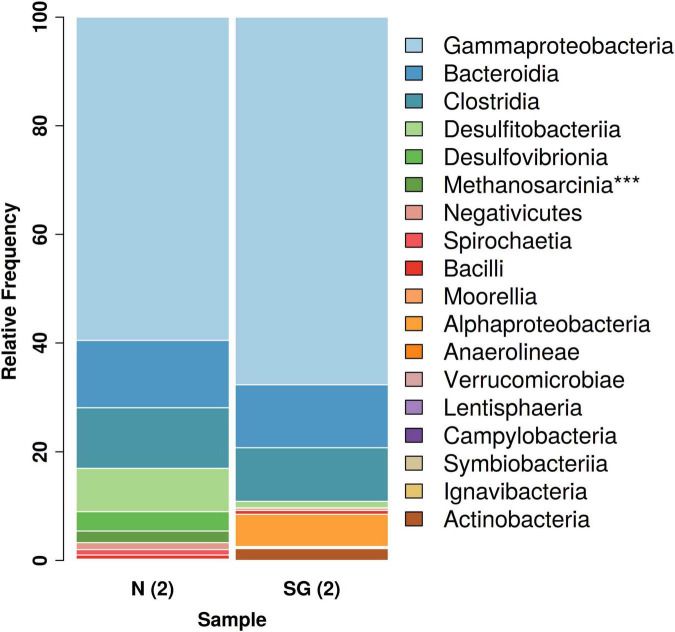
Overview of the total bacterial diversity in the two different inocula: distribution of major taxa (%) at class level (***Archaea).

The NGS analysis revealed the presence of archaeal communities in N(2) with a percentage of 2% of the total relative frequencies. These bacteria consisted mostly of members of the methanogenic genus *Methanosarcina*. The same genus was found in the inoculum SG(2) with an extremely low frequency of 0.038% (Figure 6).

**FIGURE 7:**
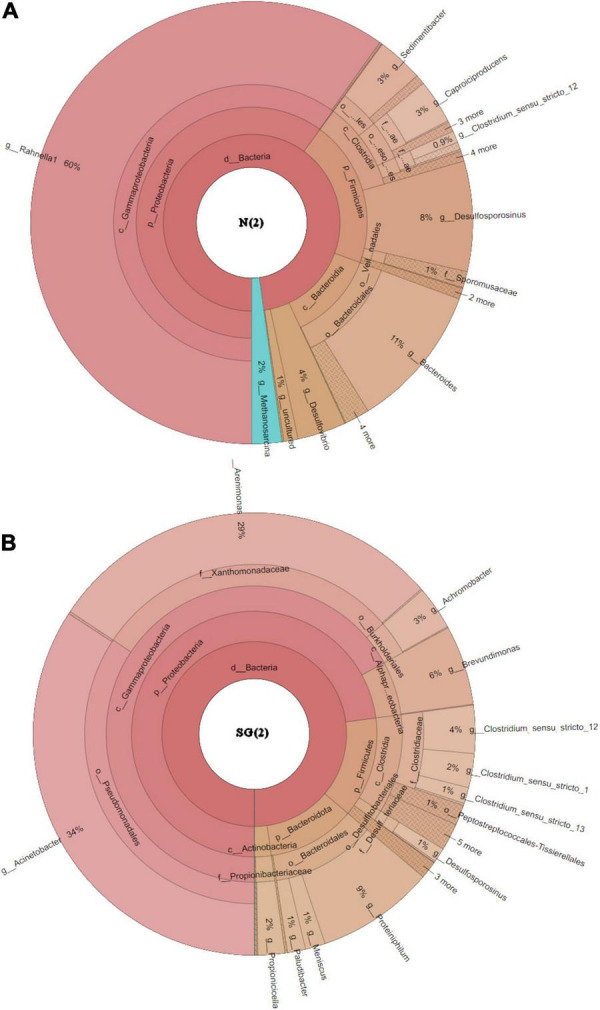
Krona plot at the genus level. **(A)** Inoculum N(2); **(B)** inoculum SG(2).

Among *Eubacteria*, a prevalence of *Gamma-Proteobacteria* followed by *Bacteroidia* and *Clostridia* was observed in both bacterial inocula. These classes dominated and had similar relative frequencies in both samples (Figure 6). Anyway, a bigger difference in the community structures was observed at the species/genus level: in the enrichment culture N(2), the most abundant genera were *Rahnella* (60% relative frequency) and *Bacteroides* (11%), followed by *Desulfosporosinus* (8%), *Desulfovibrio* (4%), and *Sedimentibacter* (3%), while *Acinetobacter* (34%), *Arenimonas* (29%), *Proteiniphilum* (9%), and *Brevundimonas* (6%) dominated in SG(2). Except for *Desulfosporosinus* and *Sedimentibacter* which were found in both samples [but in low percentage in SG(2)], the other most abundant genera appear to be specific for each inoculum examined (Figure 7).

#### Relationship Between Bacterial Communities and Chemical Characteristics of Sediment Samples

Purely for descriptive purposes, without statistical significance, we reported graphs (Supplementary Figure 5) showing different values of inoculum biodiversity indices in correspondence of higher or lower chemical elements concentrations in the sediments they come from (Table 2).

Chao1 and Shannon indices based on the ASVs number were the highest for the inoculum N(2), coming from sediment less contaminated by heavy metals and with the highest values of S_tot_. On the contrary, Simpson index was the highest in SG(2), enriched from sediment with the heaviest metal contamination.

**FIGURE 8:**
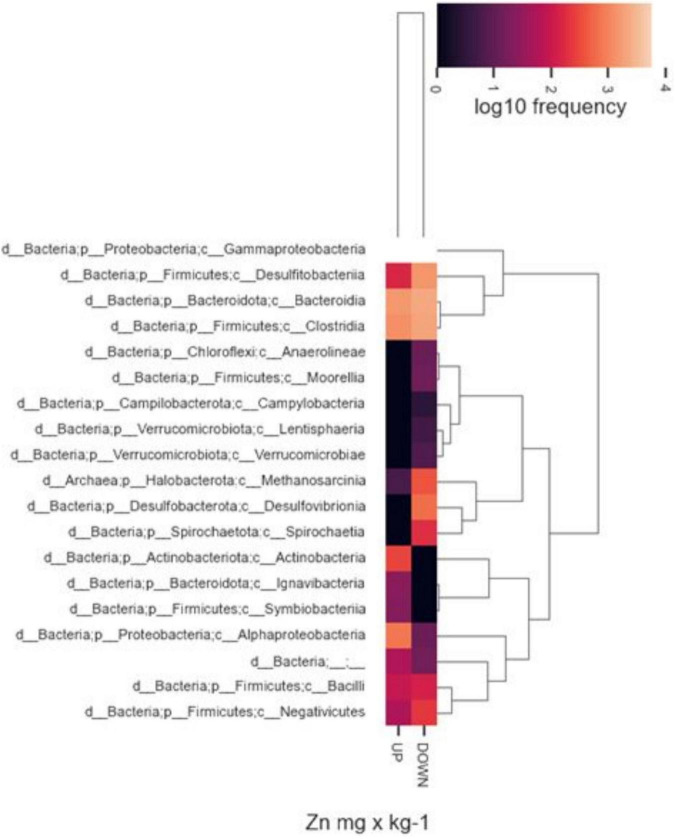
Heatmaps of relative abundance of ASVs, classified to class level using taxonomic profiling, associated with highest or lower Zn concentrations. Up, highest values; Down, lowest values.

In addition, we observed differences in taxon relative abundances at diverse levels of heavy metal contamination. Albeit in a not statistically significant way, the presence of specific microbial populations was associated with higher or lower concentrations of heavy metals in the sampling sites. By way of example, Figure 8 represents the abundance of different taxonomic classes with respect to major or minor Zn values found in the two samples of origin. In general, a high abundance of *Gammaproteobacteria*, *Bacteroidia*, and *Clostridia* was observed in both inocula regardless of sediment metal content, while other classes, resulted to be completely absent, or present with low frequencies, at lower heavy metal content (for instance, *Desulfovibronia*, *Spirochaetia*, and *Methanosarcina*). On the contrary, specific taxonomic classes were found only in sediment with higher metal content (i.e., *Actinobacteria*, *Ignavibacteria*, *Symbiobacteria*).

#### PICRUSt-Based Functional Prediction of the Community

Using PICRUSt2 analysis based on 16S rRNA sequence data, we predicted the enzyme inventories related to the major biogeochemical processes of the enriched communities. The analysis showed a total of 1,758 ECs, mostly present in both samples although with different relative abundances (Supplementary Table 2). For both inocula, five most abundant ECs predicted by PICRUSt2 were mostly housekeeping enzymes (Supplementary Figure 6).

Anyway, to find out more details on potential bio-precipitation processes, we focused the attention on enzymes that might be involved in sulfate metabolism and metal resistance. With regard to the first investigated process, an enrichment of enzymes involved in sulfate reduction, including adenylyl sulfate reductase (EC 1.8.99.2), dissimilatory sulfite reductase (EC 1.8.99.5), ATP sulfurylase (EC 2.7.7.4), and adenylyl-sulfate kinase (EC 2.7.1.25), was observed for both inocula, but with a greater abundance of most of them in the N(2) inoculum (Supplementary Table 3). Moreover, functions related to SO_4_^2–^ assimilation were generally predicted as more abundant than those related to SO_4_^2–^ dissimilation (Supplementary Table 3). Concerning the tolerance of bacteria to the high concentration of contaminants, enzymes participating in heavy-metal resistance, reduction, or transport for As, Cu, Zn, Cd, etc., and enzymes involved in oxidative stress response were also found (Supplementary Table 3).

**FIGURE 9:**
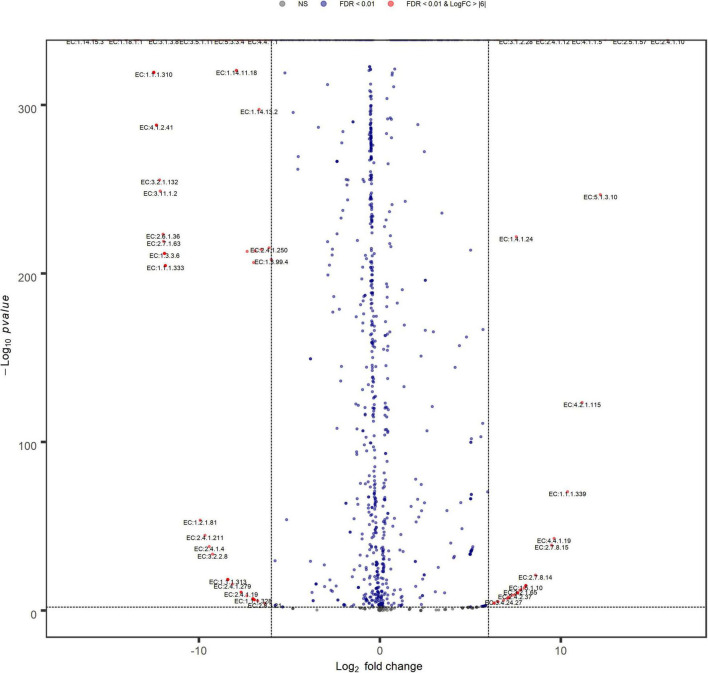
Volcano plot showing EC fold changes. Red dots represent EC relative abundances that are significant between SG(2) (left) and N(2) (right) with FDR < 0.01 and Log FC > |6|.

Volcano plot analysis (Figure 9) indicated a high similarity between inoculum metabolic functions, with few significant differences between their EC relative abundances, that do not include enzymes involved in sulfate metabolism. Anyway, changing filtering criteria (Log FC > |4|), the greater abundances of the main EC involved in sulfate reduction, higher in N(2) than SG(2), become significant (data not shown).

The analysis clearly indicated that enriched microbial populations were genetically equipped for dissimilatory sulfate reduction and heavy metal resistance.

## Discussion

The key result of our work was the selection of two different bacterial inocula, from sediments of two mine-affected streams, with similar and very satisfactory efficiency in sulfate and Zn removal from highly polluted river water. We hypothesize that the excellent heavy metal removal performances obtained for inocula selected from Naracauli N(2) and San Giorgio sediments SG(2), very different in their microbial structure and composition, are due to the selective pressure exerted by the high concentration of contaminants on the bacterial communities from which they were selected. Indeed, although each inoculum represents a small part of the microbial community residing in the sediment sample it comes from, their richness and diversity were relatively high [100 bacterial taxa in N(2) and 73 in SG(2)], not showing a complete dominance of very few species but revealing an adequate diversity even though they are enrichment cultures (values of Simpson index from 0.63 to 0.78, at the genus level). Therefore, for each inoculum, we found several different species, enriched in the medium specific for the cultivation of SRB, involved in the bio-precipitation processes. This microbial diversity could be the result of a long-term adaptation process to an ancient contamination in the polluted environments. In fact, many studies show that, while a short-term toxic impact of pollutants usually reduces the microbial diversity, chronic pollution can shape and select resilient bacterial communities with unique and rich diversity patterns, in which microorganisms are well adapted to contaminated environments over decades (Bourceret et al., 2016; Thomas et al., 2020). Therefore, the inocula selected in this study represent two distinct communities of microorganisms, adapted to different environmental conditions, but both able of using pollutants in their metabolism and tolerate them by detoxification mechanisms.

It is worth noting that the enriched cultures were both able to grow and to reduce sulfate with Zn concentrations higher than the tolerance limits reported in the literature for SRB. In fact, several studies affirm that high concentrations of heavy metals, and in particular of Zn, are one of the main factors limiting the use of SRB in bioremediation processes of highly polluted waters. About the toxicity of Zn, Radhika et al. (2006) and Azabou et al. (2007) reported concentrations exceeding 150 and 210 mg/l of Zn, respectively, as lethal for SRB, while more recent studies, referring to microbial communities of poly-extremophiles and acidotolerant/acidophilic SRB, moved the bacterial tolerance to Zn to the new limit of 450–500 mg/l (Sánchez-España et al., 2020; van der Graaf et al., 2020), observing a null or limited SRB activity in correspondence to higher values (van der Graaf et al., 2020). In the bio-precipitation experiments, the two selected inocula responded favorably despite the extreme Zn concentration of 500 mg/l in the Rio Irvi contaminated water, not only showing a high tolerance of their bacterial cells to this toxic condition but also allowing the complete removal of Zn from solution. The inoculum N(2) appeared to be more efficient in removing Fe in undiluted SRI water than SG (2), probably because less inhibited by high concentrations of Zn.

These results agree with the suggestion of many authors to stimulate bioremediation processes using SRB consortia obtained from mine-impacted sediments, containing bacteria previously exposed to contamination, and hence with more tolerance to these pollutants (Johnson and Hallberg, 2005; Castillo et al., 2012). Indeed, bacteria well adapted to extreme environmental conditions adopt known survival strategies, for instance responding to stress during their biomass growth by forming extracellular polymeric substances on which Zn could adsorb, and thus become less bioavailable to harm later generations of cells (White and Gadd, 2000). Another bacterial process that can contribute to reduce Zn from solutions could be its biosorption on the cell wall surface, by producing Zn-sulfide spherical aggregates in close relationship with bacterial cells and extracellular organic polymers (Jalali and Baldwin, 2000). However, these tolerance mechanisms helping to reduce toxic effects seem to inhibit the metabolic activity of SRB as well (Utgikar et al., 2002). In our experiments, although there was evidence of biofilm production and presence of bacteria cells in contact with ZnS spheroids (Figure 5 and Supplementary Figure 2), mineralogical evidence attested that the metabolic activity was not slowed down, allowing bacterial sulfate reduction to completely remove Zn in solution.

Framboidal metal sulfides are long time known in the geological records (Wilkin and Barnes, 1997, and references therein). They are attributed to SRB activity hosted in adequate sedimentary traps. It is actually recognized that microbial control deeply impacts the crystal growth of metal sulfide formation and affects the relative solubilities of pyrite and sphalerite (Moreau et al., 2004). The wetland-based environmental system attracted the interest of the scientific community because they are effective in the abatement of metals. Under wetland conditions, Labrenz et al. (2000) found a formation of spherical aggregates consisting of few-nanometer-diameter particles of sphalerite and associated cells ascribable to *Desulfobacter and Desulfobacterium* spp. Church et al. (2007) found ZnS spherical aggregates associated with the activity of *Desulfosporosinus* and *Desulfitobacterium*. Gammons and Frandsen (2001); Diez-Ercilla et al. (2014), and Sánchez-España et al. (2020) found spherical aggregates of ZnS in wetland systems confirming that spherical aggregates are a common morphology for ZnS formed under SRB activity. In our study, we found spherical aggregates of ZnS (Figure 4), and tubular structures in laboratory experiments (Figure 5). There are still few studies that allow to identify molecular-scale processes ruling metal sulfide authigenic formation. There is a large debate on how bacteria can control or induce biomineralization processes (Weiner and Dove, 2003; Moreau et al., 2004; and references therein). The tubular structures having a size of few microns shown in Figures 5A,D indicate that biomineralization occurs all around the cell wall. Thus, we argued that biomineralization driven by both N(2) and SG(2) consortia is likely due to an extracellular control. N(2) and SG (2) consortia comprise different strains with different metabolic functions whose effect can also depend on the quality and quantity of the molecules released at the interface between cell wall and water. Figure 5C and Supplementary Figure 3 show that tubular structures of Zn (bio)precipitates formed in 1:5 diluted Naracauli water appear to be thicker than those shown in Figure 5A. Accordingly, we previously found that both shape and size of tubules in Zn biomineralization depend also on experimental conditions (Medas et al., 2012). Thus, a further study will be conducted to better understand the biomineralization process.

Considering the similar efficiencies and performances of the two analyzed inocula, we aimed to compare their microbial taxonomic compositions looking for possible similarities in the networks of metabolic interactions among species. Interestingly, the analysis at class level, with the prevalence of *Gamma-Proteobacteria*, *Bacteroidia*, and *Clostridia*, equally distributed in both bacterial inocula, showed a convergence of the two communities. On the contrary, at species level, since the two microbial inocula do not share taxa except for *Clostridium sensu stricto*, biodiversity reflected a remarkable local characterization. Although we can refer only to the enriched communities selected in our inocula and not to the whole microbial communities of sampling sediments, we speculate that, as observed by Sprocati et al. (2014), the convergence at phylum and class levels could be related to the same pressure exerted by heavy metals, while composition in species could depend more on specific geographical and environmental parameters.

The microbial composition of the two selected inocula, as well as the environmental parameters of the sediments they come from, were quite different, so we could not carry out a statistical comparison to obtain correlations between abundances of individual taxa and the chemical parameters. Anyway, it was evident that spatial distance and different environmental conditions of the sampling sites in themselves contributed to the observed differences in species composition of the two inocula.

Inoculum NGS analysis showed sequences closely related to SRB classified in neutrophilic taxa, i.e., *Desulfovibrionaceae* and *Desulfosporosinus*, whose presence is also found in natural acidic environments due to the presence of microniches or to the existence of acidotolerant strains (Sánchez-Andrea et al., 2014). Nevertheless, it is noteworthy that the most abundant genera found in our samples do not belong to the traditional SRB groups: *Rahnella*, a facultative aerobe as well as biosurfactant-producing and heavy metal-resistant genus (Govarthanan et al., 2017), was dominant in N(2), followed by obligate anaerobes *Bacteroides*, which are known to be proficient in degrading large molecular compounds and in reducing Fe through fermentative processes in anaerobic conditions (Xia et al., 2019). The *Rahnella* genus was already identified and used to immobilize heavy metals in contaminated soils of mining areas (Zhao et al., 2019), but to the best of our knowledge, this is the first study in which it appears as a dominant taxon in a mine-impacted freshwater environment. The coexistence of *Desulfovibrio* sp. and *Bacteroides* sp., as observed in inoculum N(2), and their cooperation for sulfate reduction were reported by Zhang et al. (2016). Also *Acinetobacter*, the most abundant genus in SG(2), was proved to be dominant in the ferric-reducing conditions or in the presence of other metals like Pb, Zn, As, and arsenate which can be immobilized by the dissimilatory of Fe-reducing bacteria present in anoxic mining-impacted sediments (Xia et al., 2019). Another abundant genus detected in SG(2) inoculum was the metal-tolerant *Arenimonas*. This aerobic and facultatively anaerobic bacterium was previously described for its ability to solidify or mineralize metal(loid)s by biosorption and to survive harsh environments (average pH 5.0) (Liu et al., 2018). The *Proteiniphilum* genus, reported as obligately anaerobic strains (Zhang et al., 2015) and frequently occurring in AMD remediation studies (Sánchez-Andrea et al., 2014), was also detected in SG(2). Together with *Desulfosporosinus*, also *Sedimentibacter* was found in both samples [but in low percentage in SG(2)]. *Sedimentibacter* sp. was already detected in sediments of the highly acidic Tinto River (Spain) (Sánchez-Andrea et al., 2012) and in benthic sediments of an abandoned mine located in Urussanga, SC (Rodrigues et al., 2019), and used to treat acid mine drainage (AMD) with high metal concentrations (Zhang et al., 2016).

Therefore, although the selected inocula are completely different as regards the bacterial composition, we hypothesize that their efficacy in bio-precipitation processes was probably due to a network of common metabolic functions of differentiated species collaborating to ensure the survival of the whole bacterial community in highly polluted environmental conditions. This common metabolic adaptability could be the reason for the effectiveness of the bio-precipitation process for both inocula, showing that different autochthonous populations obtained in extreme conditions can be equally effective with respect to their performances. A common feature of N(2) and SG(2) was the low relative abundance of the most known SRB genera, acid-tolerant, anaerobic, Fe-, and sulfate reducers, *Desulfovibrio* and *Desulfosporosinus*. As reported by Xia et al. (2019), the presence of SRB (*Desulfobulbus*, *Desulfosporosinus*, and *Desulfovibrio*) may have been decreased during incubation, due to the possible reduction of sulfates in solution. In any case, although the composition in microbial species that we observed is representative of a specific moment of the biomineralization process (at the end of the period of Zn reduction), it cannot be excluded that these taxa were not very abundant in the beginning of the experiment. Indeed, many studies reiterate that low abundances of these SRB can sustain high rates of sulfate reduction, when fermentative bacteria such as *Clostridium* and highly effective syntrophic relationships are present (Pester et al., 2010; Rodrigues et al., 2020). In both our samples, a high abundance of *Clostridia* class was observed and *Clostridium sensu stricto* species was the only taxa shared between the two inocula. Previous works suggested the *Clostridium* genus as a SRB because it is strictly linked to the production of H_2_S and to biotreatment processes (Rodrigues et al., 2020). NGS analysis seems to provide new insights into the bacterial taxa involved in the sulfate reduction process as also suggested by results obtained in recent studies using molecular microbiological methods (Rodrigues et al., 2019; Zhou et al., 2020). Nowadays, it is commonly known that, although SRB have been studied for more than a century, it is only with the novel high-throughput technologies that it was possible to increase the success in identifying different novel taxa involved in the sulfate-reducing process and to obtain detailed information on their ecological roles and functions (Rodrigues et al., 2019).

In order to deepen the metabolic profiling and to investigate the genomic inventories related to sulfate metabolism and other processes of the enriched inocula, a predictive functional analysis based on 16S rRNA gene sequences using PICRUSt2 software was performed. We focused our research on particular enzymes that may be involved in dissimilatory sulfate metabolism, in the metal tolerance/transporter of As, Fe, Cu, Zn, Co, etc., and in oxidative stress response, observing that, although the microbial composition of the two samples was site-specific, the metabolic functions of interest appeared to be similar between the two inocula, indicating that different species compositions contribute to analogous metabolic outcomes to yield functional redundancy. However, considering the relative abundances of the analyzed EC, predicted functions related to SO_4_^2–^ assimilation and dissimilation seem to be more abundant in the N(2) inoculum.

Although PICRUST analysis is an indirect method to estimate microbial functions, results obtained in other works showed that predictions of metabolic microbial profiles strongly agree with results from shotgun metagenomics (Raes et al., 2021). However, despite the limitations that should be considered with this predictive analysis, our results confirmed that the enriched bacterial populations are both genetically equipped for sulfate reduction processes and heavy metal tolerance.

## Conclusion

Our research provided insights into the bioremediation potential of microbial inocula selected from highly resilient and well-adapted bacterial communities of mine-contaminated river sediments. The results suggest that pollution is a selective pressure which enhances microorganisms with metabolic capacities to tolerate and transform the contamination. This study was the first report on the selection and enrichment of sulfate-reducing microbes from sediments of the rivers San Giorgio and Naracauli (SW Sardinia), identified using a NGS analysis approach.

However, it has to be acknowledged that results obtained here describe a snapshot of the inocula microbial composition, structure, and functional potential, relative to the specific moment of the observed bio-precipitation process in which Zn was completely removed from solution. Future mineralogical, geochemical, and microbiological analyses about the kinetic and performance evolution of biomineralization processes induced by the inocula obtained in this work will offer a better understanding of the link between their genetic potential and activity and will provide deeper insight into ecological and evolutionary questions that significantly could help in the development of new bioremediation methods to reduce contaminant dispersion and for metal recovery.

## Data Availability Statement

The datasets presented in this study can be found in online repositories. The names of the repository/repositories and accession number(s) can be found in the article/Supplementary Material.

## Author Contributions

GDG coordinated the research activity. GDG, ARS, PP, CA, and FT contributed to the conception and design of the study. GDG, PAM, SN, NR, and SV collected core samples. PP and FT performed microbiological experiments and statistical analysis. DF and NR performed XRD and SEM analysis. PP, ED, DM, and MRM performed chemical analysis and data elaboration. PP, FT, and CA wrote the first draft of the manuscript. ED, DF, PAM, DM, and GDG wrote sections of the manuscript. All authors contributed to manuscript revision, read, and approved the submitted version.

## Conflict of Interest

The authors declare that the research was conducted in the absence of any commercial or financial relationships that could be construed as a potential conflict of interest.

## Publisher’s Note

All claims expressed in this article are solely those of the authors and do not necessarily represent those of their affiliated organizations, or those of the publisher, the editors and the reviewers. Any product that may be evaluated in this article, or claim that may be made by its manufacturer, is not guaranteed or endorsed by the publisher.
