# Supplementary material for: Microbial Diversity of Bacteria Involved in Biomineralization Processes in Mine-Impacted Freshwaters
Source: Front Microbiol. 2021 Nov 22;12:778199. doi: 10.3389/fmicb.2021.778199 (PMC8645857; doi:10.3389/fmicb.2021.778199)
Supplement: Supplementary file 1 [file Data_Sheet_1.zip › Data Sheet 1.docx]

**Supplementary Material**

**Supplementary Figure 1.** mineralogical composition of core sediments collected from Rio Naracauli stream sediments. Qtz = quartz; Fsp = feldspar; Phy = phyllosilicates; Sul = sulphides; Cal = calcite; Ap = apatite; Sd = siderite; Jar = jarosite; Cer = cerussite; Ank = ankerite; Smt = smithsonite; Gt = goethite.





**Supplementary Figure 2.** SEM images of bacteria, microbial biofilms and mineral precipitates obtained for SG inoculum in the three different liquid media: (A): medium 1; (B): medium 2; (C): medium 3. (D) Optical microscope images of SG inoculum in standard Postgate B medium


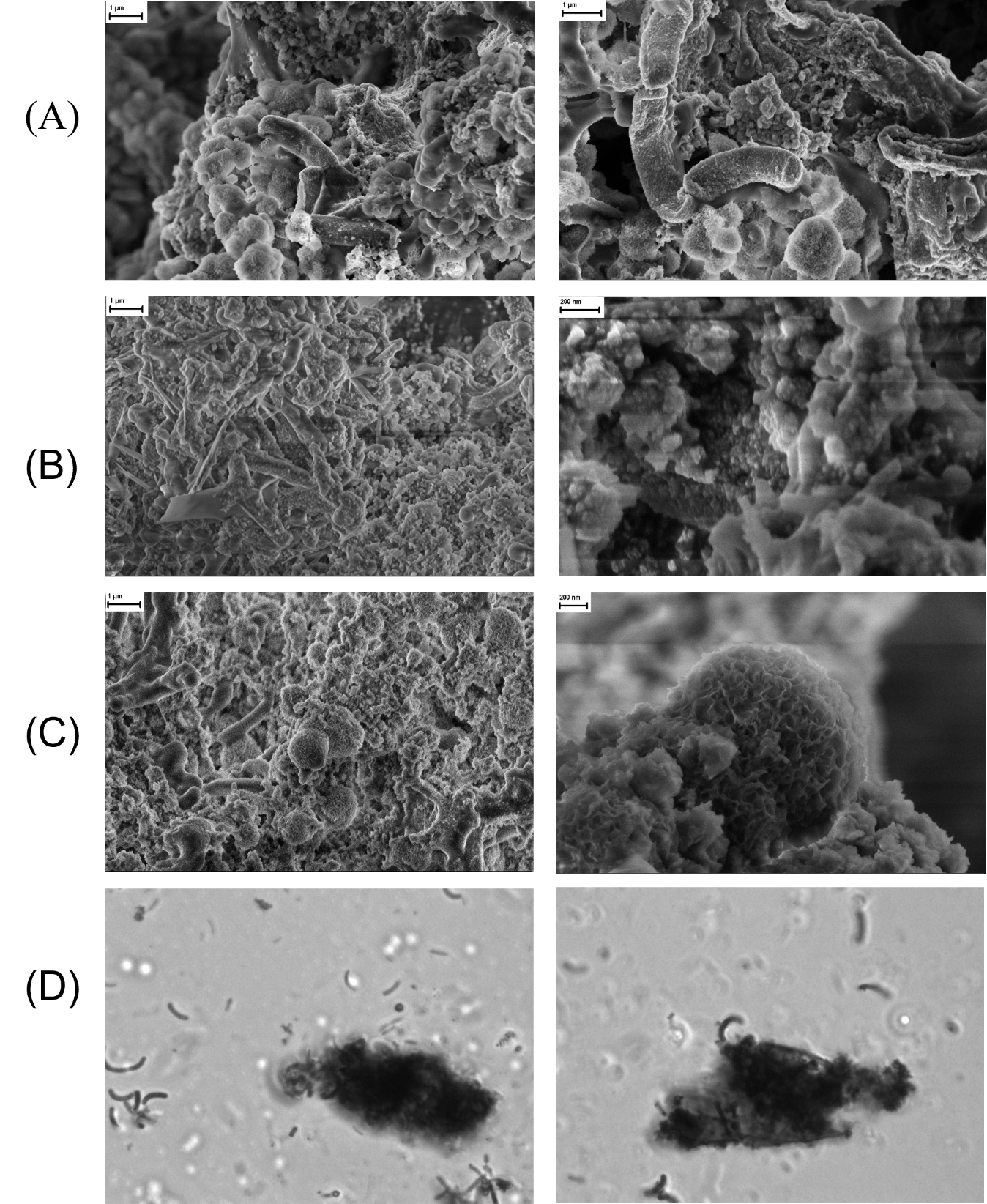


**Supplementary Figure 3.** Scanning electron microscopy (SEM) analysis: A) BSE images of BSE images of bioprecipitates recovered from experiment performed with inocula selected from Rio Naracauli core sediments and diluted Rio Irvi water (sample N(2) Irvi(1:5)) and B) its EDS spectrum.


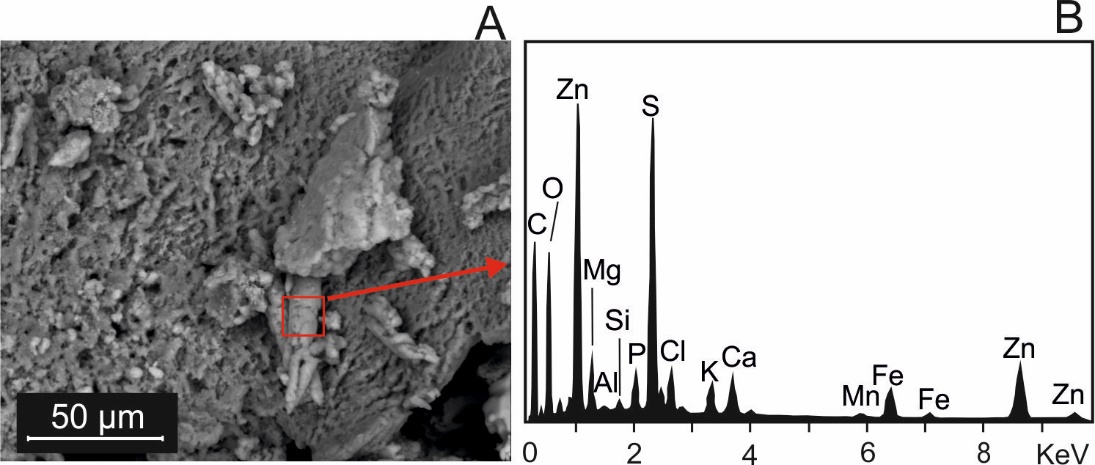


**Supplementary Figure 4:** Rarefaction curves of the two samples tending to the saturation plateau.


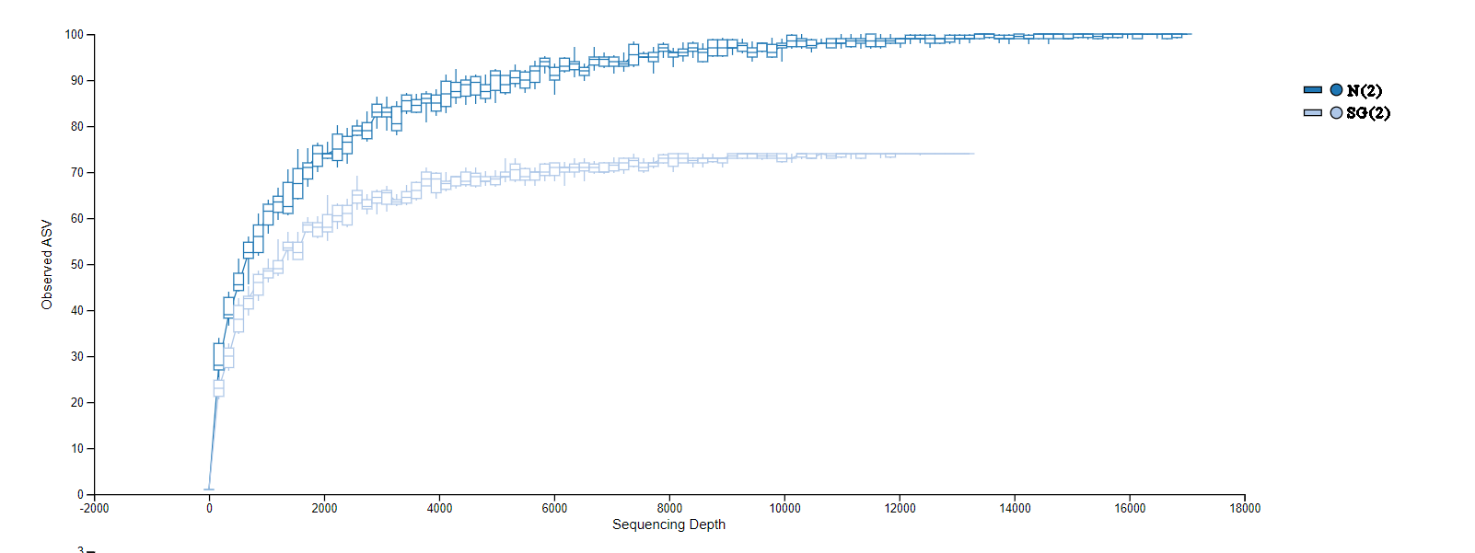


**Supplementary Figure 5:** Box-plots illustrating alpha diversity indices (Chao1 index, Shannon diversity and Simpson index) for the two inocula enriched from sediments with different chemical elements concentrations.


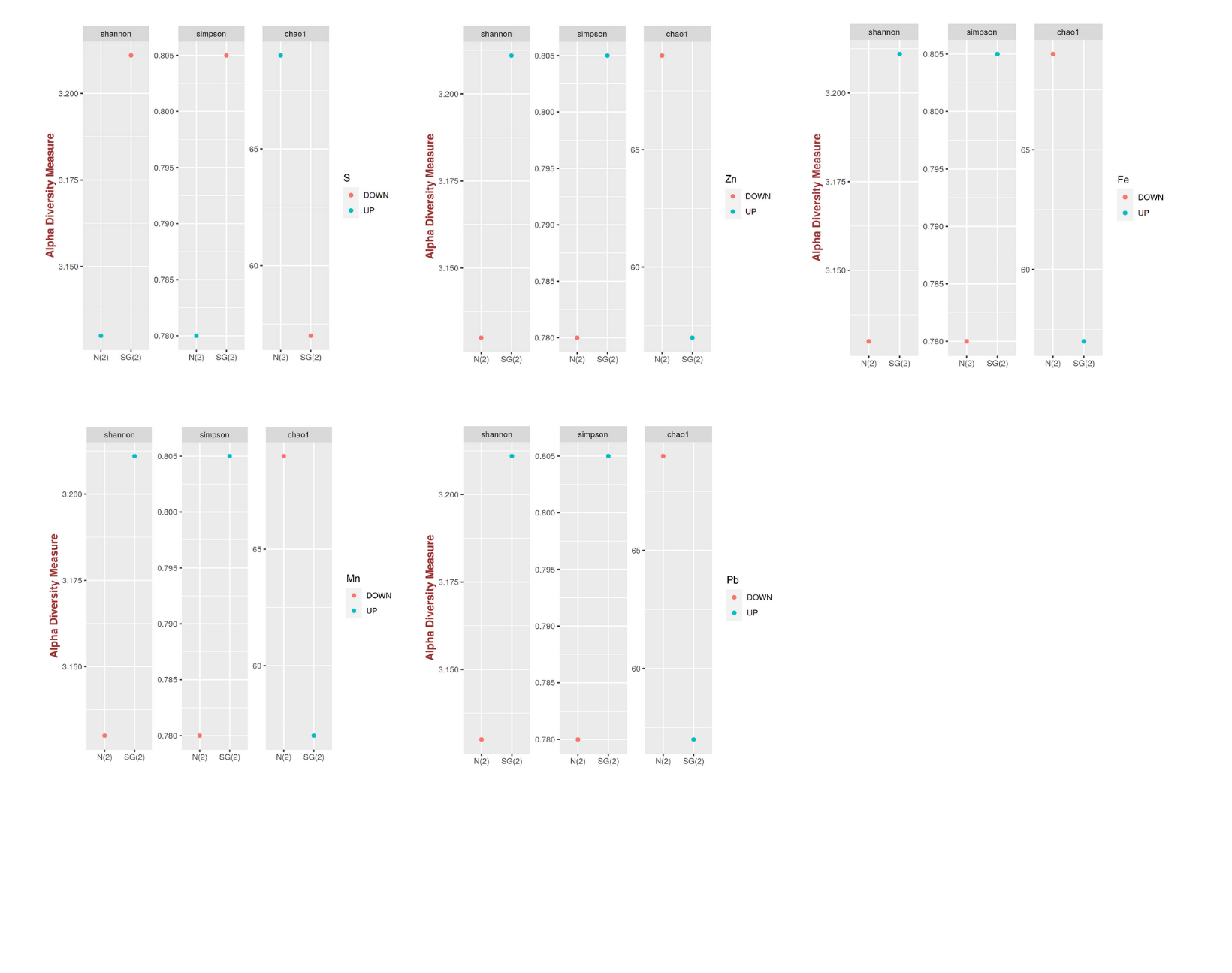


**Supplementary Figure 6:** The 5 most abundant enzymes predicted by PICRUSt2

**
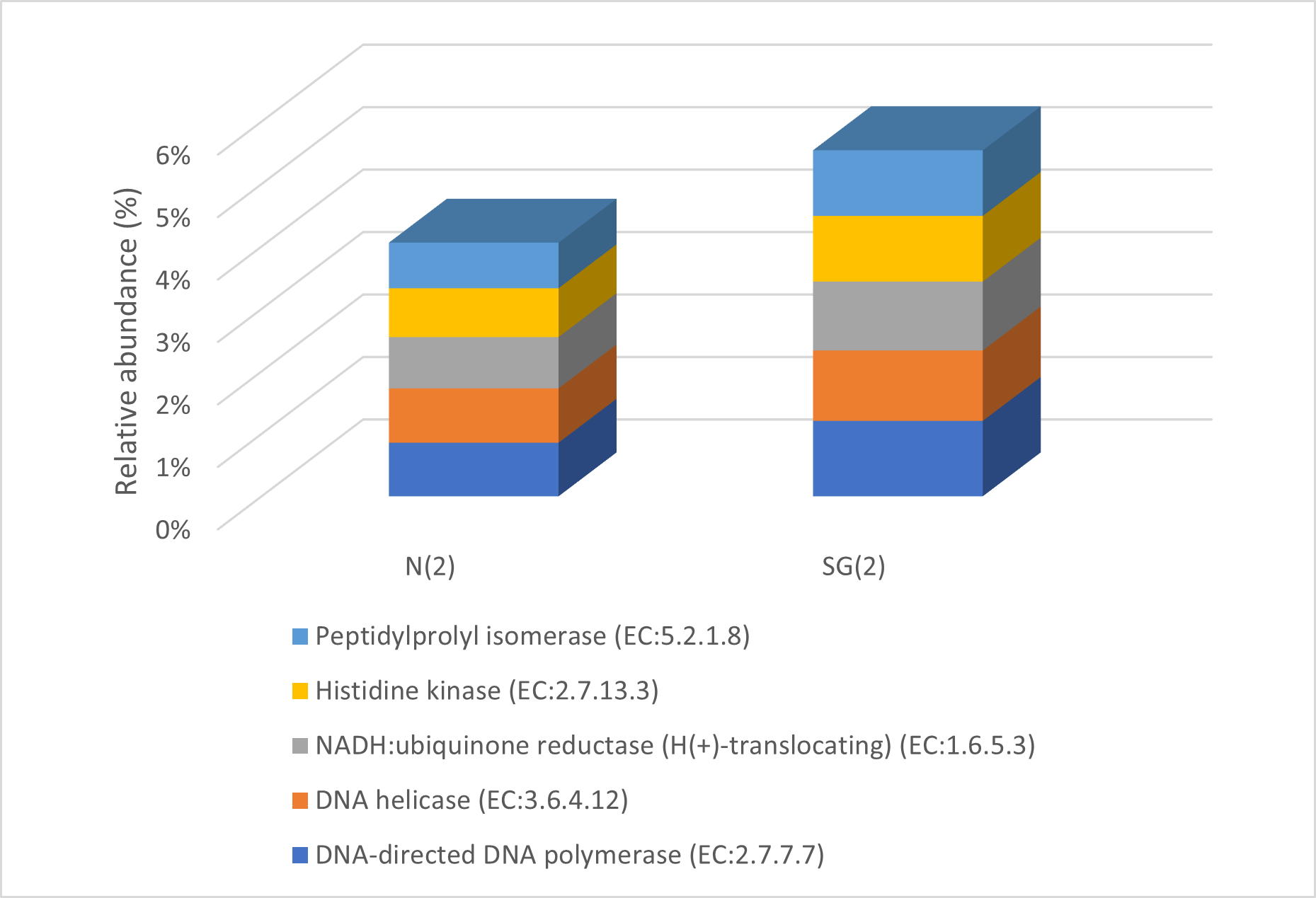
**

**Supplementary Table 1:** Range values of discharge, pH and selected chemical species detected in filtered (0.4 µm) waters of Rio Naracauli, Rio San Giorgio and Rio Irvi.

|  |  | **Rio Naracauli^1^** | **Rio San Giorgio^2^** | **Rio Irvi^3^** |
| --- | --- | --- | --- | --- |
| **Discharge** | L/s | 0.4 – 35 | 17 – 37 | 21 – 29 |
| **pH** |  | 7.6 – 8.4 | 7.7 – 8.3 | 4.8 – 6.3 |
| **Zn** | mg/L | 2.0 – 64 | 0.05 – 2.5 | 760 – 860 |
| **Fe** | mg/L | < DL | < DL – 47 | 130 – 220 |
| **Mn** | μg/L | 0.2 – 660 | 7.7 – 1120 | 57000 – 66000 |
| **Cd** | µg/L | 55 – 490 | 0.03 – 31 | 1600 – 2000 |
| **Pb** | µg/L | 1.2 – 90 | 0.6 – 16 | 4 – 440 |
| **Ni** | µg/L | 0.07 – 90 | 1.7 – 2.6 | 2000 – 3100 |
| **Cu** | µg/L | < DL – 90 | < DL – 5.8 | < DL |
| **Co** | µg/L | 0.45 – 15.9 | 0.4 – 0.96 | 1400 – 1700 |
| **Mo** | µg/L | 0.7 – 1.4 | 0.21 – 0.89 | < DL |
| **SO_4_** | mg/L | 290 – 850 | 230 – 620 | 2900 – 3400 |
| ^1^De Giudici et al. (2014), ^2^De Giudici et al. (2017), ^3^De Giudici et al. (2018)  DL_Fe_ = 0.2 mg/L; DL_Cu_ = 1 µg/L; DL_Mo_ = 10 µg/L. | | | | |

**Supplementary Table 2 (EXCEL file attached):** Relative abundances of Enzyme Commission numbers (ECs) present in the two samples

**Supplementary Table 3:** List of enzymes involved with sulphate reduction, metal resistance and stress responce predicted by PICRUSt2

|  |  |  | Abundances | |
| --- | --- | --- | --- | --- |
| EC Number | Description | Interest | N(2) | SG(2) |
| EC:1.11.1.15 | Peroxiredoxin | Stress response | 45853.76 | 22538.76 |
| EC:1.15.1.1 | Superoxide dismutase | Stress response | 17935.02 | 19022.91 |
| EC:1.15.1.2 | Superoxide reductase | Stress response | 3267.21 | 2292.19 |
| EC:1.16.1.1 | Mercury(II) reductase | Metal resistance/transport | 0 | 894 |
| EC:1.16.1.9 | Ferric-chelate reductase (NADPH) | Metal resistance/transport | 10745 | 0 |
| EC:1.16.3.2 | Bacterial non-heme ferritin | Metal resistance/transport | 15411.54 | 2587.11 |
| EC:1.20.4.1 | Arsenate reductase (glutaredoxin) | Metal resistance/transport | 29216.55 | 13189.41 |
| EC:1.8.1.2 | Assimilatory sulfite reductase (NADPH) | Assimilatory sulfate reduction | 22239 | 8525.4 |
| EC:1.8.1.9 | Thioredoxin-disulfide reductase | Stress response | 22586.13 | 13463.37 |
| EC:1.8.4.8 | Phosphoadenylyl-sulfate reductase (thioredoxin) | Assimilatory sulfate reduction | 12155.83 | 2004.54 |
| EC:1.8.7.1 | Assimilatory sulfite reductase (ferredoxin) | Assimilatory sulfate reduction | 185.59 | 481 |
| EC:1.8.99.2 | Adenylyl-sulfate reductase | Dissimilatory sulfate reduction | 4082.08 | 1909.96 |
| EC:1.8.99.5 | Dissimilatory sulfite reductase | Dissimilatory sulfate reduction | 2421.76 | 102.5 |
| EC:2.7.1.25 | Adenylyl-sulfate kinase | Assimilatory sulfate reduction | 12552.24 | 5722.73 |
| EC:2.7.13.3 | Histidine kinase | Metal resistance/transport | 144205.93 | 81582.98 |
| EC:2.7.7.4 | Sulfate adenylyltransferase | Assimilatory/Dissimilatory sulfate reduction | 25229.21 | 14454.11 |
| EC:3.6.3.16 | Arsenite-transporting ATPase | Metal resistance/transport | 1980.9 | 1244.78 |
| EC:3.6.3.2 | Magnesium-importing ATPase | Metal resistance/transport | 21518.83 | 464.67 |
| EC:3.6.3.27 | Phosphate-transporting ATPase | Metal resistance/transport | 17077.54 | 9987.56 |
| EC:3.6.3.29 | Molybdate-transporting ATPase | Metal resistance/transport | 13717.08 | 2964.9 |
| EC:3.6.3.3 | Cadmium-exporting ATPase | Metal resistance/transport | 17572.75 | 5931.11 |
| EC:3.6.3.4 | Cu(2+)-exporting ATPase | Metal resistance/transport | 3625.33 | 6184.17 |
| EC:3.6.3.5 | Zinc-exporting ATPase | Metal resistance/transport | 17572.75 | 5931.11 |
| EC:3.6.3.54 | Cu(+) exporting ATPase | Metal resistance/transport | 14851.88 | 15019.86 |
| EC:5.3.4.1 | Protein disulfide-isomerase | Metal resistance/transport | 10817 | 6837.4 |
